# HUSH, a Link Between Intrinsic Immunity and HIV Latency

**DOI:** 10.3389/fmicb.2019.00224

**Published:** 2019-02-12

**Authors:** Ghina Chougui, Florence Margottin-Goguet

**Affiliations:** ^1^Inserm, U1016, Institut Cochin, Paris, France; ^2^CNRS, UMR8104, Paris, France; ^3^Université Paris Descartes, Sorbonne Paris Cité, Paris, France

**Keywords:** restriction factors, latency, reservoirs, HIV auxiliary proteins, epigenetic control, HUSH, Vpx, Vpr

## Abstract

A prominent obstacle to HIV eradication in seropositive individuals is the viral persistence in latent reservoir cells, which constitute an HIV sanctuary out of reach of highly active antiretroviral therapies. Thus, the study of molecular mechanisms governing latency is a very active field that aims at providing solutions to face the reservoirs issue. Since the past 15 years, another major field in HIV biology focused on the discovery and study of restriction factors that shape intrinsic immunity, while engaging in a molecular battle against HIV. Some of these restrictions factors act at early stages of the virus life cycle, alike SAMHD1 antagonized by the viral protein Vpx, while others are late actors. Until recently, no such factor was identified in the nucleus and found active at the level of provirus expression, a crucial step where latency may take place. Today, two studies highlight Human Silencing Hub (HUSH) as a potential restriction factor that controls viral expression and is antagonized by Vpx. This Review discusses HUSH restriction in the light of the actual knowledge of intrinsic immunity and HIV latency.

Human Immunodeficiency Virus is responsible for the Acquired Immunodeficiency Syndrome. Since its start in the early 1980s, HIV pandemic claimed about 35 million lives. In 2017, an estimated 36.9 million people were living with HIV worldwide. Despite a global decline, thanks to the increased and early access to antiretroviral therapy, in 2017, 1.8 million people were newly infected and almost 1 million died of AIDS-related illnesses (Global HIV and AIDS Statistics, 2018 fact sheet). Though many battles have been won, as HIV is no longer a death sentence but a manageable chronic illness in several parts of the world, the war for HIV eradication remains to win. Statistics above mirror the crucial need for further understanding of HIV biology and its interaction with the host, to anticipate future challenges in HIV infection control and eradication.

Lately, one major issue of HIV eradication was raised by HIV latency and precisely the persistence of reservoir cells in infected individuals, despite antiretroviral therapy. Indeed, these cells harboring integrated but silent viruses were found to be a source for viral rebound following treatment interruption. Another major field of interest is the study of restriction factors that counteract the virus at different step of its life cycle and shape intrinsic immunity. Today the characterization of the Human Silencing Hub (HUSH) complex has created a link between these two domains of interest. Here, we discuss current knowledge about restriction factors, molecular mechanisms governing HIV latency, and finally strategies to address latency. The common thread of these three parts will be a focus on HUSH.

## Hush, a Molecular Player of Intrinsic Immunity?

**FIGURE 1:**
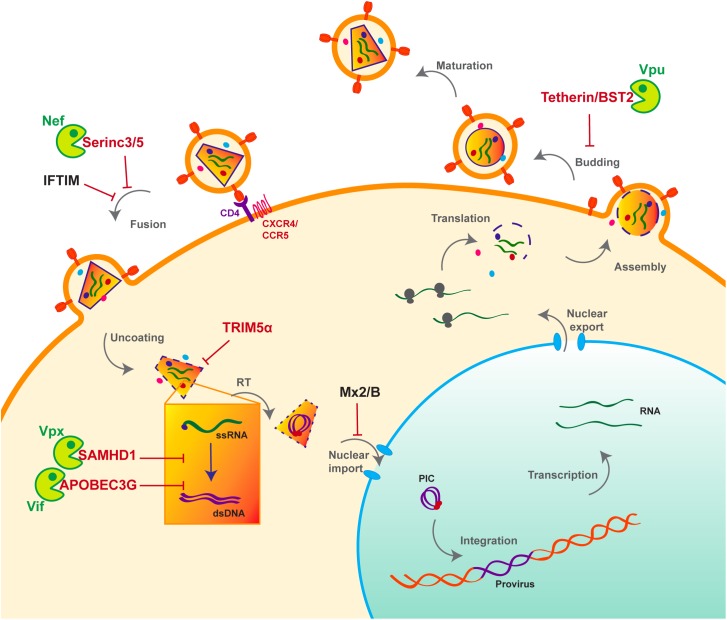
Host antiviral factors and viral agonists. Following virion adhesion to the cellular receptor and coreceptor, HIV enters the cell through fusion of its envelope with the cellular membrane. The capsid is therefore destabilized and Reverse transcription (RT) is triggered, allowing the synthesis of a double stranded DNA (dsDNA) using the viral single stranded RNA (ssRNA) as a matrix. Viral DNA along with other viral proteins including Vpr, Matrix (MA) and Integrase (IN) form the pre-integration complex (PIC), which is imported into the nucleus thanks to Nuclear Localization Signals (NLS) (Brown et al., 1987). This nuclear import step is critical as it allows HIV to infect non-dividing cells, in contrast to other retroviruses such as HTLV, which requires nuclear membrane destabilization. After viral DNA integration, viral genes are transcribed by the cellular machinery and the viral protein Tat; various forms of spliced RNAs are exported from the nucleus for translation. Unspliced RNAs are escorted by the viral protein Rev and will constitute the viral genome of the newly assembled virion. Finally, immature viral particles bud at the membrane packaging both viral and cellular factors. Constitutively expressed antiviral restriction factors are in red, others are in black. Restriction factors might be antagonized by viral auxiliary proteins, shown in green, some being specific to HIV-1/SIVcpz (Vpu), other to HIV-2/SIVmac/SIVsmm (Vpx).

HIV life cycle can be separated in two phases: early and late (Figure 1). The early phase extends from viral adhesion to integration, whereas late stages start from transcription to budding and release of the new particle (Figure 1). Throughout the whole replication process, HIV faces several barriers set up by the host cell to protect its integrity. Indeed, various cellular factors are able to detect viral elements and trigger an antiviral response. It is the case of sensors like cyclic GMP–AMP synthase (cGAS) and IFNγ-Inducible protein 16 (IFI16), which can detect HIV viral cDNA released in the cytoplasm and induce a signaling cascade resulting in the induction of interferon stimulated genes (ISGs) (Gao et al., 2013; Jakobsen et al., 2013; Lahaye et al., 2013). HIV replication is inhibited by interferon (IFN) stimulation in a cell- type dependent manner, the antiviral response predominantly relies on type I interferon (IFN-I), including interferon-α (IFN-α). Myxovirus resistance 2 (MX2), an IFN-α inducible factor, contributes to the early block of HIV replication, by preventing nuclear import of the viral cDNA (Figure 1) (Goujon et al., 2013; Kane et al., 2013; Liu et al., 2013). Moreover, antiviral factors can incorporate within the viral particle, as for IFN-induced transmembrane (IFITM) proteins. IFITM 1, 2, and 3, which are also found on the viral membrane, can hinder viral entry by interfering with viral fusion (Figure 1) (Lu et al., 2011; Compton et al., 2014; Tartour et al., 2014).

### Restriction Factors

**FIGURE 2:**
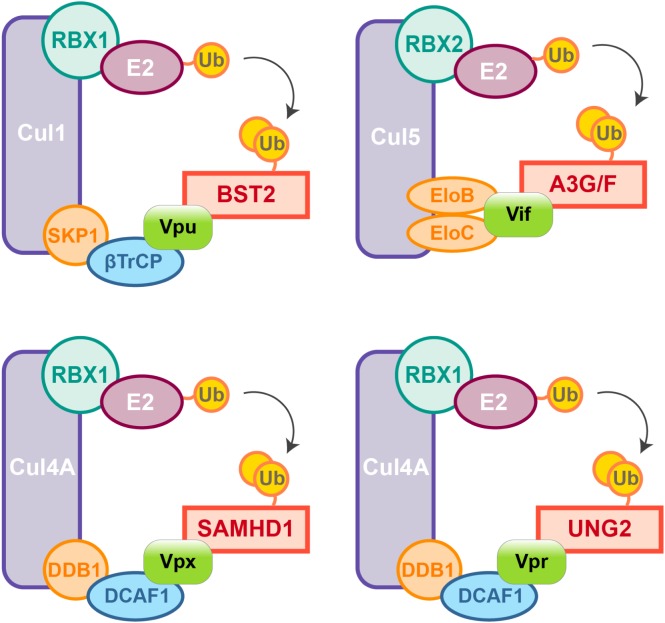
HIV auxiliary proteins hijack the ubiquitin-proteasome pathway. HIV auxiliary proteins hijack different Cullin-RING Ligases (CRLs). CRLs share the same architecture, they are built on a Cullin scaffold (purple) binding a RING-box protein (RBX1 or 2, green) along with adaptor proteins (blue and orange) which allow substrate recognition. Vif induces APOBEC3G and F degradation by usurping the E3 ubiquitin ligase Cul5, Vif mimicks the adaptor SOCS (not represented) and directly interacts with the adaptors Elongin B and C. In contrast, HIV-1 Vpu hijacks the E3 ubiquitin ligase Cul1 through interaction with the βTrCP adaptor. HIV-2/SIVsmm Vpx uses the same strategy and binds the adaptor DCAF1 to recruit the E3 ubiquitin ligase Cul4A and induces proteasomal degradation of SAMHD1. Cul4A-DDB1^DCAF1^ ubiquitin ligase is also hijacked by HIV-1 Vpr to target the Uracyl DNA Glycosylase (UNG2) (Ahn et al., 2010).

However, even before the establishment of the interferon response, a group of cellular factors have the capacity to directly interrupt diverse stages of the viral replication cycle (Figure 1). These antiviral factors constitute the cellular intrinsic defense and in order to escape from their control, lentiviruses evolved ingenious tools, namely auxiliary proteins (Figure 1, 2). Though dispensable for viral replication, at least *in vitro*, auxiliary or accessory proteins have the ability to specifically inactivate a small group of these cellular antiviral factors, termed restriction factors. The notion of “restriction factor” was first mentioned in the 70s, when the protective effect of Friend virus susceptibility protein 1 (Fv1) against MLV was discovered (Lilly, 1970; Pincus et al., 1971; Rowe et al., 1973). Years later, primate lentiviruses were found sensitive to such antiviral factors, yet the exact definition of a restriction factor is still debated. Indeed, diverse cellular factors are able to block HIV replication as well as other viruses, a set of criteria was therefore proposed to distinguish restriction factors from resistance factors (Harris et al., 2012; Malim and Bieniasz, 2012; Doyle et al., 2015).

Generally, at least two features are shared by restriction factors: (i) cell-autonomous antiviral activity, i.e., the ability to inhibit viral replication without the need of cell to cell communication; (ii) direct interaction with the pathogen, which in turn can escape restriction either by changing the interaction surface or inducing the targeted inactivation of the restriction factor. Due to this direct interaction and as a result of host-pathogen co-evolution, most restriction factors evolved under positive selection, i.e., the spread of protein-altering mutations. Moreover, as they are tightly linked to the cellular defense mechanism, some restriction factors can be IFN-inducible. To date only five restriction factors have been identified: Apolipoprotein B mRNA-Editing enzyme, Catalytic polypeptide-like 3G (APOBEC3G) (Sheehy et al., 2002; Conticello et al., 2003; Harris et al., 2003; Mangeat et al., 2003; Zhang et al., 2003), Tripartite motif-containing protein 5 (TRIM5α) (Stremlau et al., 2004, 2006; Black and Aiken, 2010), Bone Stromal Tumor protein 2 (BST2)/Tetherin (Neil et al., 2008; Van Damme et al., 2008), Sterile Alpha Motif domain and HD domain-containing protein 1 (SAMHD1) (Hrecka et al., 2011; Laguette et al., 2011) and Serine Incorporator 3 and 5 (Serinc3/5) (Matheson et al., 2015; Rosa et al., 2015; Usami et al., 2015).

Apolipoprotein B mRNA-editing enzyme, catalytic polypeptide-like 3G and SAMHD1 are both active at early stages of HIV replication (Figure 1). SAMHD1 blocks the reverse transcription step by decreasing the pool of intracellular deoxynucleoside triphosphates (dNTP) (Goldstone et al., 2011; Hrecka et al., 2011; Laguette et al., 2011; Baldauf et al., 2012; Descours et al., 2012; Lahouassa et al., 2012), whereas the cytidine deaminase APOBEC3G, incorporates into the viral particle and induces C to U hyper-mutations on the viral DNA during reverse transcription, hence resulting in viral genes alteration (Sheehy et al., 2002; Conticello et al., 2003; Harris et al., 2003; Mangeat et al., 2003; Zhang et al., 2003). However, thanks to its auxiliary proteins, HIV is able to evade these two restriction factors (Figure 1). Indeed, Vif prevents the packaging of APOBEC3G by inducing its polyubiquitination, resulting in its subsequent proteasomal degradation (Sheehy et al., 2002, 2003; Mariani et al., 2003; Yu et al., 2003; Mehle et al., 2004) (Figure 1, 2). On the other hand, Vpx, which is unique to HIV-2 and not encoded by HIV-1, uses the same strategy to trigger SAMHD1 proteasomal degradation (Hrecka et al., 2011; Laguette et al., 2011) (Figure 1, 2). APOBEC3G and SAMHD1 both harbor signs of positive selection as a result of direct contact with viral proteins (Sawyer et al., 2004; Laguette et al., 2012). Although both SAMHD1 and APOBEC3G are constitutively expressed in many cell types, their expression can also be induced by IFN stimulation (Lafuse et al., 1995; Li et al., 2000; Sarkis et al., 2006; Tanaka et al., 2006; Stopak et al., 2007; Riess et al., 2017).

Alike APOBEC3G and SAMHD1, Tetherin/BST2, which is active at late stages of viral replication (Figure 1), can also be induced by IFN stimulation. BST2 prevents the release of viral particles from the producer cell by retaining virions on the cell surface. HIV-1 auxiliary protein Vpu helps the virus evade BST2 by down-regulating and sequestrating it away from viral budding sites (Neil et al., 2008; Van Damme et al., 2008). As a consequence of this viral antagonism, BST2 was shown to have evolved under positive selection (Gupta et al., 2009; McNatt et al., 2009; Lim et al., 2010).

Tripartite motif-containing protein 5 is a E3-ubiquitin ligase which can also be IFN-induced (Sakuma et al., 2007; Carthagena et al., 2008). It inhibits viral replication by binding the capsid and accelerating its proteasomal degradation, thus inducing premature uncoating which prevents reverse transcription (Stremlau et al., 2004, 2006; Black and Aiken, 2010). Interestingly and in contrast to the factors described above, TRIM5α is not targeted by a viral protein. Instead, retroviruses evade its restriction through capsid mutation, hence altering the interaction surface and avoiding recognition by TRIM5α (Hatziioannou et al., 2006; Kamada et al., 2006). This also explains why rhesus monkey but not human TRIM5α inhibits HIV replication (Stremlau et al., 2004, 2006; Black and Aiken, 2010). TRIM5α is therefore an important barrier for cross-species transmission, which also evolved under positive selection (Sawyer et al., 2005; Tareen et al., 2009).

Based on their features and compared to the previously described factors, Serinc 3 and 5 are intriguing. Serinc 3 and 5 are both packaged into new virions and inhibit viral fusion with the target cell (Matheson et al., 2015; Rosa et al., 2015; Usami et al., 2015). HIV-1 uses Nef to evade Serinc 3/5 and induces its down-regulation. Nef dramatically increases virions infectivity by preventing Serinc3/5 incorporation through its removal from cell surface (Matheson et al., 2015; Rosa et al., 2015; Usami et al., 2015). Serinc 3/5 is not up-regulated by IFN and despite its interaction with Nef, no sign of molecular arm race were found in Serinc3/5, meaning no recent virus-host co-evolution (Murrell et al., 2016).

### The Molecular Arm Race: Adapt to Thrive

Interaction with pathogens, including viruses, profoundly shaped the evolutionary history of human intrinsic immunity and particularly that of restriction factors, which very often are in direct contact with these threats (Duggal and Emerman, 2012). Restriction factors represent a selective pressure on viruses but once these viruses mutate to escape restriction, adapted cellular factors then provide a fitness advantage to their host. Therefore, a competition for continuous adaptation arises between the virus and its host, termed “the molecular arms race.” Such genetic conflicts between host and viral proteins frequently results in events of “positive selection,” meaning an excess of non-synonymous mutations (dN) compared to synonymous mutations (dS). Positively selected sites often correspond to the interaction interface between host and viral factors, such sites are found in TRIM5α, APOBEC3G, Tetherin/BST2, and SAMHD1 (Sawyer et al., 2004, 2005; McNatt et al., 2009; Lim et al., 2010; Laguette et al., 2012).

Nonetheless, due to constrains related to their cellular role, positive selection is not the only strategy used by hosts’ factors to cope with viral escape, other events such as polymorphism, gene duplication and various innovations including the presence of different isoforms are observed, alike APOBEC3 family which counts seven paralogs with cytosine deaminase activity. Although less active against HIV than APOBEC3G, APOBEC3D, F, and H show restricting activities but only APOBEC3F and one haplotype of APOBEC3H were found counteracted by Vif (Wiegand et al., 2004; Liu et al., 2005; Binka et al., 2012). On the other hand, despite lower fidelity of their reverse transcriptase resulting in higher mutation rates, viruses also evolve under tight constrains, a consequence of their small genome size and overlapping open reading frames (ORFs). Some viral proteins can also have several roles and interact with both viral and cellular factors. Furthermore, in order to antagonize a single restriction factor, in addition to direct binding of the target, a viral protein may require interaction with a secondary host factor. It is the case of viral-induced proteasomal degradation of cellular factors, which relies on the hijacking of cellular ubiquitin ligases, especially Cullin-RING Ligases (CRLs) in the case of HIV (Figure 2). For instance, Vpx requires the DDB1-Cullin4-associated factor 1 (DCAF1) as an adaptor to hijack the Cul4A ubiquitin ligase (Figure 2) (Le Rouzic et al., 2007; Sharova et al., 2008; Srivastava et al., 2008).

Viral protein R (Vpr), a paralog of Vpx usurps the same ubiquitin ligase but does not degrade SAMHD1. Instead Vpr has different functions among them the mysterious induction of cell-cycle arrest in dividing cells (He et al., 1995; Jowett et al., 1995; Re et al., 1995; Le Rouzic et al., 2007; Wen et al., 2007; Sharova et al., 2008; Srivastava et al., 2008).

### SAMHD1 Antagonism by Vpx

**FIGURE 3:**
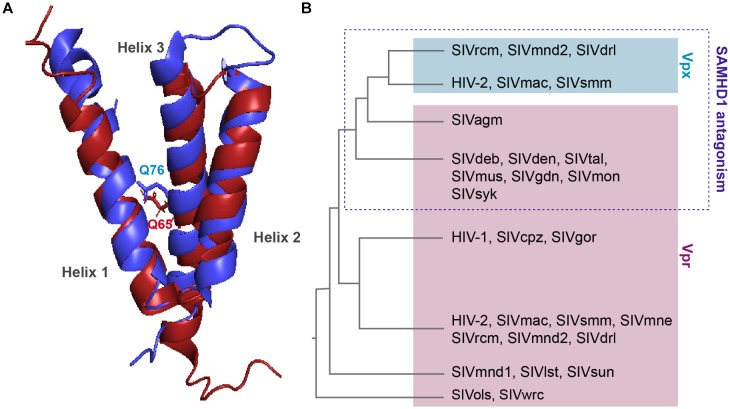
Structure comparison and origin of Vpr and Vpx. **(A)** Structural similarities. Alignment of the 3D PDB structures of HIV-1 Vpr (red) and SIVsmm Vpx (blue). Three α-helix structures are separated with loops and two flexible regions at both Nter and Cter ends. One residue critical for DCAF1 binding is represented on each structure, Q65 on HIV-1 Vpr and Q76 on SIVsmm Vpx. In contrast to Vpr, Vpx is stabilized by a zinc finger motif [PDB:1M8L (Morellet et al., 2003), SIVsmm Vpx (PDB: 4cc9 (Schwefel et al., 2014)]. **(B)** Schematic representation of a Vpx/Vpr phylogenic tree. Vpx are in the blue box, Vpr are in the pink, based on (Lim et al., 2012).

Viral protein R and X (Vpx) were identified in the early 90’s, they are incorporated into the viral particle and thus present at early stages of viral replication (Henderson et al., 1988; Kappes et al., 1988; Cohen et al., 1990a,b). These two proteins share the same three-helix bundle structure and are both able to bind DCAF1 through residues within the third α-helix (Figure 3A).

*Vpr* and *vpx* genes are thought to originate from complex events of duplication and/or recombination of one common precursor gene (Sharp et al., 1996; Tristem et al., 1998), but in contrast to *vpr* which is found in all primate lineages, its paralog, *vpx*, is only present in two lineages: HIV-2/SIVsmm/SIVmac and SIVrcm/SIVmnd2 from red-capped monkeys and from mandrill (Figure 3B) (Beer et al., 2001; Hu et al., 2003).

Interestingly, despite divergence of their sequences, *vpx* genes from different lineages cluster together away from their homologous *vpr* genes from the same lineage (Figure 3B). Furthermore, though these two proteins hijack the same ubiquitin ligase, Vpr and Vpx bear different functions. Vpr has the mysterious ability to induce cell-cycle arrest in dividing cells, which contribution to viral replication remains unknown, whereas Vpx induces SAMHD1 degradation, hence relieving a block on reverse transcription. However, some Vpr from lineages lacking Vpx are exceptions as they possess both of these functions, alike Vpr from SIVagm and SIVsyk, respectively, from African green monkey and Syke’s monkey (Stivahtis et al., 1997; Lim et al., 2012).

In contrast to the mystery surrounding the role of Vpr, Vpx function during viral replication is rather well understood, at least partly. This 12–16 kDa protein is incorporated into the viral particle and expressed by only two lineages as stated above. Although Vpx seems dispensable for viral replication in lymphocytic cell lines (Yu et al., 1988; Hu et al., 1989), *in vivo* its deletion was reported to negatively impact SIV spread and kinetics in monkeys (SIVsmm, SIVmac, and SIVmne from pig-tailed macaques) (Gibbs et al., 1995; Hirsch et al., 1998; Belshan et al., 2012). Loss of Vpx was also shown to drastically impair viral replication at early stages in activated peripheral blood mononuclear cells (PBMCs), primary lymphocytes and, with even greater effects in monocyte-derived macrophages (MDMs) (Guyader et al., 1989; Kappes et al., 1991; Yu et al., 1991; Gibbs et al., 1994; Kawamura et al., 1994; Park and Sodroski, 1995). Moreover, viral transduction with both SIVmac and HIV-1-derived lentivectors was increased following Vpx delivery through virus-like particle (VLP) in MDMs and in monocyte-derived dendritic cells (MDDCs) (Goujon et al., 2006), such effect was, however, absent in activated primary T cells. The same positive impact of Vpx was further observed on HIV-2/SIVsmm and HIV-1 full length viruses and was shown to depend on the proteasome, precisely on the hijacking of the Cul4A-DDB1^DCAF1^ ubiquitin ligase (Goujon et al., 2007; Fujita et al., 2008a; Sharova et al., 2008; Srivastava et al., 2008; Bergamaschi et al., 2009). Vpx activity was found critical for the reverse transcription step in MDMs, in which the lack of Vpx strongly reduced viral DNA synthesis, a phenomenon observed with Feline Immunodeficiency virus (FIV) and MLV as well (Goujon et al., 2007; Fujita et al., 2008a; Sharova et al., 2008; Srivastava et al., 2008; Bergamaschi et al., 2009). Altogether, these observations demonstrated the existence of an early block on viral replication in myeloid cells, which was not specific to HIV-2/SIVsmm viruses, but counteracted by Vpx through ubiquitination. Vpx was therefore expected to inactivate, *via* the proteasome, a restriction factor active at reverse transcription and specific of myeloid cells. This model was finally confirmed after the identification of the Vpx target SAMHD1 (Hrecka et al., 2011; Laguette et al., 2011), which was later found also active in quiescent T cells (Baldauf et al., 2012; Descours et al., 2012).

Several Vpx mutants have been described as reviewed in Schaller et al. (2014). Vpx Q76A or Q76R and K77A, which no longer bind DCAF1, were found both unable to induce SAMHD1 degradation (Srivastava et al., 2008; Bergamaschi et al., 2009; Hrecka et al., 2011). Wild-type Vpx and the Vpx Q76A mutant were shown to rescue HIV-1 infection in IFN-treated MDDCs (Pertel et al., 2011), independently from dNTP levels and after completion of reverse transcription (Reinhard et al., 2014). These results suggested the existence of another Vpx strategy to antagonize SAMHD1 or a second IFN-inducible target of Vpx. In addition, Vpx deletion was reported to impair viral replication in activated PBMCs and lymphocytes (Guyader et al., 1989; Kappes et al., 1991; Yu et al., 1991; Akari et al., 1992), in which SAMHD1 is thought to be inactive. Such replication defect due to Vpx deletion was also observed in HSC-F cells that express undetectable levels of SAMHD1 (Ueno et al., 2003; Fujita et al., 2008b). Finally, Vpx from SIVmnd2/SIVrcm were recently reported to counteract a SAMHD1-independent restriction in human resting CD4+ T cells (Baldauf et al., 2017). Taken together, these observations indicate the existence of additional Vpx targets, unrelated to SAMHD1.

### HUSH Antagonism by Vpx and Vpr Lentiviral Proteins

**FIGURE 4:**
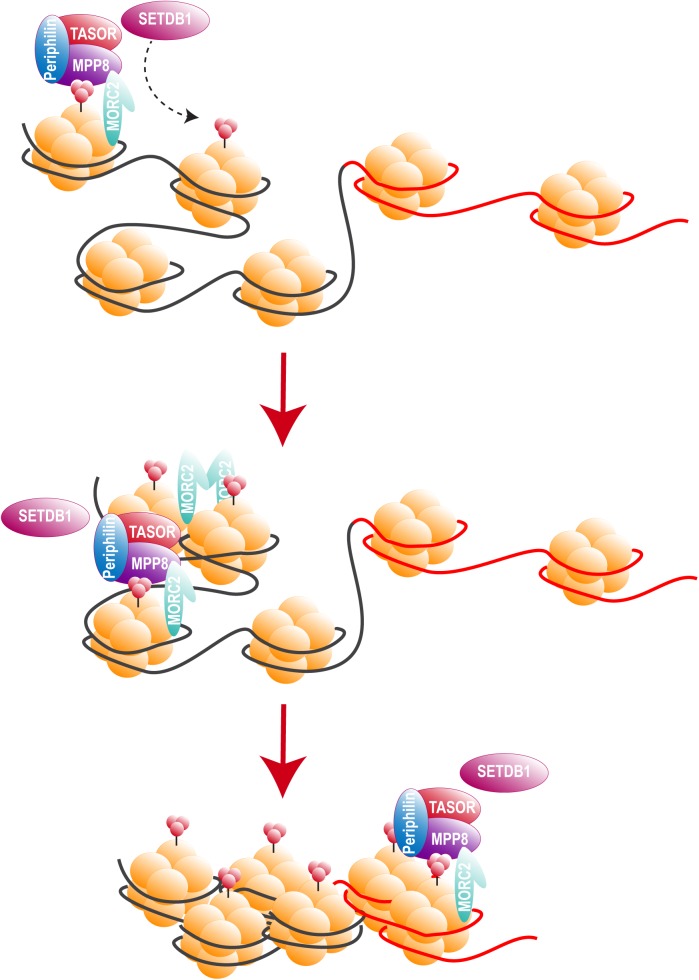
The HUSH complex. HUSH complex comprises three members: MPP8, able to bind H3K9me3 through its chromodomain (Kokura et al., 2010; Chang et al., 2011; Li et al., 2011), TASOR and Periphilin, which functions within the complex are not yet determined. HUSH complex localizes on H3K9me3 rich regions and allows the spread of heterochromatin in cooperation with at least SETDB1, resulting in the silencing of cellular genes (black) and integrated transgenes (red). HUSH silencing activity also relies on MORC2. MORC2 dimerization is thought to allow nucleosome remodeling, though MORC2 ATPase activity (Li et al., 2012; Tchasovnikarova et al., 2017; Douse et al., 2018, 2).

Two recent studies, including ours, identified HUSH as a cellular complex inactivated by Vpx (Chougui et al., 2018; Yurkovetskiy et al., 2018). The TASOR/FAM208A component of HUSH appeared first as c3orf63 in an siRNA screen for HIV antiviral factors (Liu et al., 2011), then in a screen for indirect chromatin reader in mouse tissues (Eberl et al., 2013). Also, in a study aimed at identifying factors involved in the epigenetic control, Harten et al. (2014) mutagenized a mouse cell line carrying a multi-copy transgene expressing GFP under the control of the human alpha globin promoter. In this mouse line, 45% of erythrocytes do not express GFP as a result of epigenetic silencing, thus Harten et al. (2014) selected mutants with an increased percentage of GFP expressing cells and generated mutant mouse lines, among which MommeD6 and MommeD20, the first mice heterozygous mutants for FAM208A. Of note, in this model, FAM208A was also found to be critical for normal mouse development (Harten et al., 2014). Then, FAM208A was renamed as TASOR for Transgene Activation SupreressOR, a member of an epigenetic complex involved in chromatin silencing, the HUSH complex (Tchasovnikarova et al., 2015). In this study, the HUSH complex was identified in a screen for factors involved in position-effect variegation (PEV), i.e., the silencing of a normally active gene as a result of its positioning into heterochromatin (Elgin and Reuter, 2013). Namely, Tchasovnikarova et al. (2015) transduced the near-haploid cell line KBM7 with a lentiviral construct expressing GFP from the spleen focus-forming virus (SFFV) promoter and then sorted the population of transduced cells with a low GFP expression (GFP^dim^). Following mutagenesis of the GFP^dim^ population and the analysis of mutant cells with an increased GFP expression (GFP^bright^), SETDB1, Family protein with sequence similarity 208A (FAM208A), Matrix Metalloproteinase-8 (MPP8) and Periphilin appeared as critical repressors of transgene expression. Tchasovnikarova et al. (2015) study found FAM208A, renamed as TASOR, to interact with both Periphilin and MPP8, forming the HUSH complex, which in turn recruited the methyl transferase SETDB1 (Figure 4). It was therefore suggested that HUSH complex could maintain transcriptional silencing by facilitating the spread of H3K9me3 marks on integrated transgenes (Figure 4).

**FIGURE 5:**
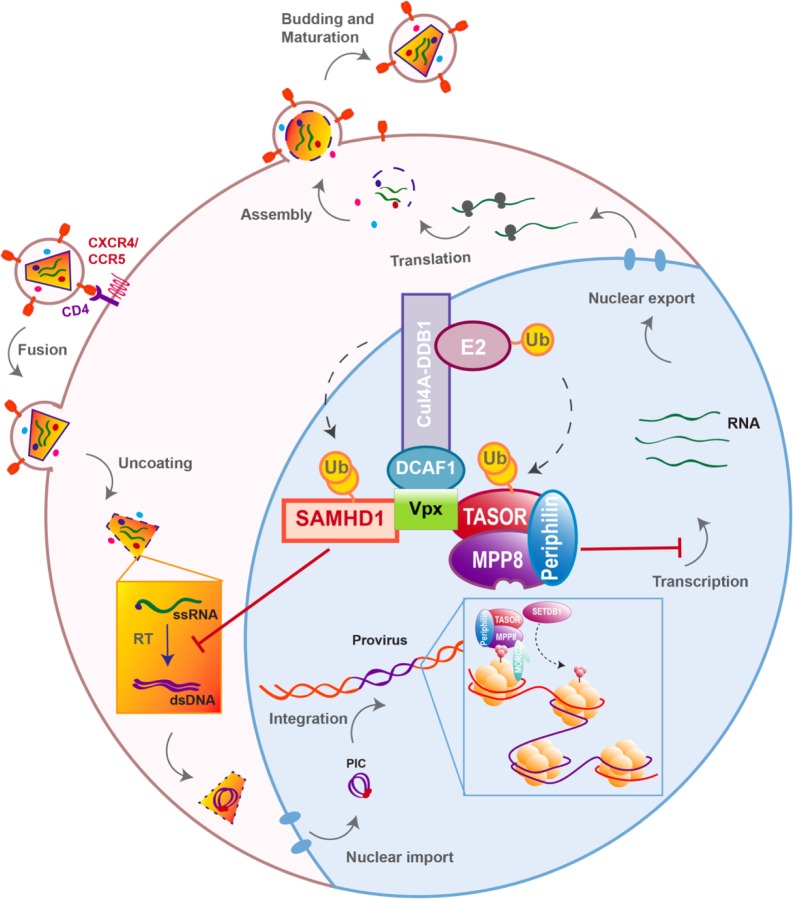
Vpx overcomes two independent blocks on viral replication. To simplify the scheme, ubiquitination and binding of the HUSH complex is represented through TASOR.

In our study, we retrieved TASOR as the second best down-regulated target of Vpx, just after SAMHD1, by performing a Stable-Isotope Labeling by Amino acids in Cell culture (SILAC) proteomic screen (Chougui et al., 2018). Tasor was also selected in a loss-of-function Vpx study focused on genes that contribute to silencing of retroviruses (Yurkovetskiy et al., 2018). Both studies further showed that Vpx interacts with HUSH and induces its proteasomal degradation through the hijacking of DCAF1, using the same mechanism as the one described for SAMHD1 antagonism. Though, HUSH antagonism by Vpx is genetically separable from SAMHD1 antagonism as revealed by a Vpx mutant defective for HUSH degradation, but still able to inactivate SAMHD1 (Chougui et al., 2018). Mimicking HUSH depletion, Vpx induces the reactivation of a latent HIV-1 provirus through H3K9me3 marks depletion in the J-Lat A1 model of latency (Chougui et al., 2018). Furthermore, HUSH depletion gives an advantage to replication-competent HIV-1 or SIVmac239-ΔVpx viruses (Yurkovetskiy et al., 2018). Altogether, these data report a new role of Vpx that counteracts HUSH, a complex active at the post-integration level (Figure 5). Interestingly, this unexpected function is both independent from SAMHD1 and unrelated to the previously described SAMHD1-independent effects of Vpx, either in IFN-treated MDDCs (Pertel et al., 2011; Reinhard et al., 2014) or in quiescent CD4+ T cells, which seems to take place early at the level of reverse transcription (Baldauf et al., 2017). These observations therefore suggest the existence of remaining Vpx targets besides SAMHD1 and the HUSH complex.

### Is HUSH a Restriction Factor?

Alike SAMHD1, HUSH complex is constitutively active in a cell-autonomous manner and is counteracted by at least one viral protein through proteasomal degradation, via the hijacking of a cellular E3 ubiquitin ligase. However, global evolutionary analysis failed to identify residues under positive-selection, as it is the case with Serinc3/5. Instead, members of the HUSH complex exist in multiple and variable isoforms as it is the case for TASOR and Periphilin (which has also undergone HERV-M insertion), such diversifications can be considered as signatures of a potential gene-conflict (Huh et al., 2006; Chougui et al., 2018).

Antagonism of HUSH complex by *vpx/vpr* genes is ancestral and precedes the birth of Vpx and SAMHD1 antagonism. It is also a species-specific function as some but not all Vpr/Vpx were able to recognize the human HUSH complex. However, testing for human HUSH recognition gives no indication on whether these inactive Vpr/Vpx maintain or not this function against HUSH complexes from their corresponding species. Indeed, although Vpx from SIVrcm-GAB1 did not degrade human HUSH complex as found in our study, Vpx from other SIVrcm strains did (NG411 and 02CM8081), as shown in Yurkovetskiy et al. (2018). In fact, we already have observed such differences in the capacity to recognize and degrade human HUSH complex when testing different Vpr from SIVagm. Here again, while Vpr from SIVagm.ver-KE.9063 and SIVagm.sab-1 degraded human HUSH, SIVagm.gri-677 did not, these results were also reproduced and extended to other SIVagm Vpr in Yurkovetskiy et al. (2018). Unfortunately, both Yurkovetskiy et al. (2018) and our study lack positive controls for the functionality of Vpr/Vpx that do not degrade human HUSH.

Overall, although the capacity of these Vpr/Vpx to recognize the HUSH complex of their corresponding species were not yet investigated, these results encourage the idea of a molecular arms race with perhaps limited possibilities of evolution for the members of the HUSH complex. Strong constrains on HUSH members may have arisen from the crucial cellular role played by this complex in maintaining genomic integrity and regulating the expression of more than 900 cellular genes (Tchasovnikarova et al., 2015). HUSH complex activity also have critical implications in developmental process as mice mutant for TASOR were found not viable beyond gastrulation (Harten et al., 2014; Bhargava et al., 2017). Moreover, besides the need to maintain interactions within the complex, cooperation with both MORC2 and SETDB1 were found required for the functioning of the HUSH complex (Tchasovnikarova et al., 2015, 2017, 2). Adding to this, HUSH complex and especially TASOR was found to collaborate with Tripartite motif-containing 28 (TRIM28) in the silencing of L1 elements (Robbez-Masson et al., 2018). The existence of numerous partners may have further reduced the possible evolutionary trajectories for members of the HUSH complex, thus the identification of critical sites under positive-selection may require beforehand isolation and analysis of precise regions within the complex that are at the interface with the viral proteins Vpx/Vpr. Of note, direct interaction between Vpx and members of HUSH has not been investigated yet, it is not excluded that cellular intermediates bridge the viral protein to the epigenetic repressor complex.

Finally, in the light of our actual knowledge, HUSH complex seems to share common features with restriction factors, especially with Serinc 3/5. Future studies will have to determine whether HUSH complex is wired to the immune system, by assessing the impact of IFN stimulation on its expression and antiviral activity.

### HIV-2 Clinical Features: Is HUSH Antagonism by Vpx a Paradox?

In contrast to HIV-1, HIV-2 is less infectious with lower rates of transmission and is restricted to West Africa. HIV-2 is less pathogenic with low plasma viremia and 86–95% of HIV-2 infected patients are considered long-term-non-progressors, whereas only 5–15% HIV-1 positive patients are (Martinez-Steele et al., 2007). Overall, HIV-2 displays a higher tendency for viral latency as reviewed in Saleh et al. (2017), thus Vpx capacity to counteract HUSH raises questions regarding the clinical outcomes of HIV-2. Indeed, one would expect HUSH antagonism by Vpx to result in a highly expressed HIV-2 instead of the weak expression observed in patients. However, HIV-2 is also able to counteract SAMHD1, APOBEC3G, and Tetherin/BST2, yet it remains a low expressed virus. Therefore, clinical features does not seem to necessarily correlate with the restriction factors a virus can overcome, as also demonstrated in a study which found no differences in SAMHD1 antagonism by Vpx alleles from HIV-2 viremic and long-term aviremic patients (Yu et al., 2013).

HIV-2 low expression may result from a stronger immunological control, as HIV-2 triggers a greater immune-response in patients than HIV-1 does, reviewed in Nyamweya et al. (2013). One could imagine the targeting and degradation of too many restriction factors by HIV-2 to be responsible for a better immune sensing. Being too sensitive to these restriction factors, HIV-2 may have evolved tools to escape their repression but with a considerable cost on its fitness i.e., an increased vulnerability to detection by the immune system.

However, such hypothesis is less likely as SIVsmm, at the origin of HIV-2, replicates intensively in its natural hosts without inducing AIDS and shows high levels of plasma viremia, which can be even higher than those observed in HIV-1 patients (Rey-Cuillé et al., 1998; Silvestri et al., 2003). Furthermore, SIVsmm infection is characterized by lower levels of immune activation than observed with HIV-2 (Silvestri et al., 2003, 2005; Sumpter et al., 2007). The immunological control of HIV-2 might therefore arise from rather a lack of adaptation of this virus to its new host, nonetheless, HIV-2 viral expression is still high enough to allow viral transmission.

### Impact of HUSH Antagonism on the Epigenetic Landscape

Overall, HUSH antagonism by Vpx raises questions over the impact of HIV-2 infection on the epigenetic landscape and subsequently on cellular genes as well as exogenous element expression. If HUSH degradation is confirmed in cells from infected patients, then it would be interesting to asses expression of genes under the control of the HUSH complex and to further evaluate its possible consequences on HIV infection and in terms of disease development. Indeed, HUSH complex is involved in the epigenetic control of more than 900 loci, with many genes belonging to the Krüppel-associated box (KRAB) domain-containing zinc-finger proteins (KZFPs) family of transcription repressors (Tchasovnikarova et al., 2015). KZFP proteins are involved in various process ranging from development to metabolism and even cancer as reviewed in Lupo et al. (2013).

## What Potential Role for HUSH in the Context of HIV Latency?

Activated CD4+ T cells are highly susceptible to HIV infection, in most cases viral replication results in the death of the infected cell. However, some infected T cells survive long enough to return to a resting state, as memory T cells. This reversible shift from activated to resting state is a normal physiological process, allowing rapid immune responses following re-exposure to an antigen. As resting state is non-permissive for viral replication, these long-lived memory T cells will bear a silent but replication-competent provirus, therefore constituting the major latent reservoir (Chun et al., 1997; Finzi et al., 1999; Chomont et al., 2009). These reservoirs are established early after infection, initiation of cART during primary infection was shown to reduce the size of these reservoirs and allow a better control of viremia following treatment interruption (Chun et al., 1998; Strain et al., 2005; Archin et al., 2012b; Buzon et al., 2014). Nonetheless, even under prolonged antiretroviral therapy, latent reservoir cells persist (Siliciano et al., 2003), forming an HIV sanctuary out of reach of conventional therapies.

### Molecular Mechanisms of Latency

Latency is a complex, multifactorial process that involves different cells, cellular subsets and the combination of several molecular mechanisms (Margolis, 2010), only three points will be discussed below: availability of cellular factors, integration site and chromatin-mediated repression.

**FIGURE 6:**
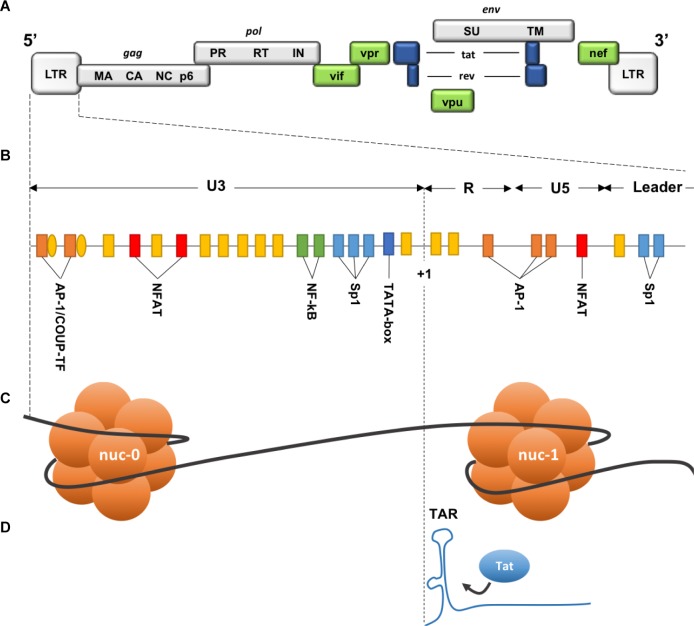
Description of the 5**′** LTR of HIV-1. **(A)** HIV genomic structure. **(B)** Binding sites for cellular factors within the 5**′** LTR. **(C)** Nucleosomes structure within the 5**′** LTR. **(D)** *Trans-*activation Response element on the neosynthesized RNA. HIV genome is bordered by two identical Long Terminal Repeats (LTR 5′ and 3′), each composed of 3 regions: U3 (Unique 3′), R (repeated), and U5 (Unique 5′). Three functional units can be identified within U3 region: (1) a modulatory element with binding sites for transcription regulators including negative regulators. (2) an enhancer containing two binding sites for NF-κB. (3) the core promoter region comprising 3 binding sites for specificity protein 1 (Sp1) and a TATA box. R region contains the transactivation response element TAR, an RNA stem-loop at the 5′ end of the neo-synthesized strand, necessary for Tat-dependent transcription and elongation block alleviation, while U5 region contains additional modulatory/regulatory sites. Nucleosome nuc-1 is located immediately after the transcription start site (+1), chromatin remodeling is required to overcome this second elongation block. (Adapted from Colin and Van Lint, 2009).

Two identical Long Terminal Repeat (LTR) sequences are located at both extremities of HIV provirus (Figure 6). U3 region of the 5′ LTR serves as a promoter recruiting cellular transcription factors and the RNA pol II. Due to the elongation block of the RNA pol II by negative factors (Wu et al., 2003), incomplete viral transcripts will be synthesized, these transcripts will, however, allow the production and accumulation of the viral *trans-*activator protein (Tat). Tat is necessary for elongation activation (Kao et al., 1987), it binds the *trans-*activation response element (TAR) (Figure 6), and subsequently recruits the positive transcription elongation factor-b (P-TEFb). This will result in a positive feedback loop and only then will the HIV transcription be efficiently activated. Thus, the triggering of HIV transcription critically relies on the host’s factors availability, some of which are inducible and dependent on the cellular state, as for NF-κB and NFAT that are found sequestrated in the cytoplasm in the absence of cellular activation signals. Stimulation of latently infected cells with phorbol myristate acetate (PMA), phytohemagglutinin (PHA) or Tumor Necrosis Factor α (TNFα), results in the reactivation of HIV transcription in several latency model cells. Low levels of transcription factors may therefore contribute to the non-permissive state of resting CD4+ T cells, along with P-TEFb levels that show a drastic increase during cellular transition from quiescent to activated state (Budhiraja et al., 2013).

Due to integrase interaction with Lens epithelium-derived growth factor (LEDGF/p75) (Llano et al., 2006; Shun et al., 2007), HIV-1 favors integration into introns of actively transcribed genes, near the nuclear pores (Schröder et al., 2002; Marini et al., 2015). Unexpectedly, the same integration pattern was observed in resting CD4+ T cells from infected patients under cART (Han et al., 2004). Both defective proviruses and transcriptional interference due to close host’s genes promoters were proposed to explain this non-productive state (Han et al., 2008; Lenasi et al., 2008). In addition, sensitivity to nearby heterochromatin may contribute to the silencing of HIV promoter. Indeed, in different cellular clones, heterogeneity of basal expression of HIV promoter depending on the integration site was observed (Jordan, 2001), and although less than 1% of integration events are thought to directly lead to latency (Schröder et al., 2002), integration within or near heterochromatin regions resulted in latent proviruses (Jordan et al., 2003; Sherrill-Mix et al., 2013; Battivelli et al., 2018). Moreover, following global activation signals, latently infected Jurkat clones (J-Lat) showed a variegated reactivation of HIV promoter and a different rate of de-activation once the signal was withdrawn, likely due to different integration sites (Jordan et al., 2003). Same position effect variegation following reactivation signals was linked to variable distances from enhancer sequences and repressive chromatin (Chen et al., 2016). Of note, regulatory elements can impact genes hundreds of kilobases away through various interactions allowed by the spatial genome organization (Gibcus and Dekker, 2013), distant interactions such as gene looping can also allow heterochromatin spreading and the silencing of active genes (Talbert and Henikoff, 2006). Even though the integration context alone is not sufficient to predict the fate of infection, it is an additional factor that contributes to both the establishment and reversal of latency.

Two nucleosomes (nuc-0 and nuc-1) form within the promoter region of the 5′ LTR of HIV provirus (Figure 6), nuc-1 is of most importance as it is located immediately downstream of the transcription start site and therefore constitute an additional elongation block (Verdin et al., 1993). To overcome this block and allow HIV-1 promoter activation, nuc-1 remodeling by SWItch/Sucrose Non-Fermentable (SWI/SNF) complex is required (El Kharroubi et al., 1998; Agbottah et al., 2006; Mahmoudi et al., 2006; Tréand et al., 2006; Rafati et al., 2011). Maintenance of this nuc-1 repressive structure was also associated with histone deacetylation, more precisely the recruitment by several transcription factors of the histone deacetylase 1 (HDAC1) (Van Lint et al., 1996; Sheridan et al., 1997; Coull et al., 2000; He and Margolis, 2002; Lusic et al., 2003). Other histone modifications are involved in HIV-1 LTR repression such as trimethylation of the Lysine 9 or 27 of the histone 3 (H3K9me3 and H3K27me3). Suv39H1, a histone lysine methyl transferase (HKMT), responsible for H3K9 trimethylation, is recruited on the viral promoter by the negative factor CTIP2. HP1 binds the H3K9me3 repressive marks and subsequently recruits additional Suv39H1, this self-sustaining loop is thought to allow the spread and maintenance of heterochromatin, hence reducing DNA access to transcription factors (du Chéné et al., 2007, 39; Marban et al., 2007). Additional HKMTs were shown to participate in HIV-1 LTR silencing, such as G9a/GLP (Imai et al., 2010) and very recently SETDB1 through its interaction with the HUSH complex (Tchasovnikarova et al., 2015).

Human Silencing Hub complex activity was demonstrated on different cellular and viral promoters including the Murine Leukemia Virus (MLV) and HIV-1 LTRs (Tchasovnikarova et al., 2015). In J-Lat cells, HUSH depletion resulted in promoter reactivation and GFP expression, following a loss of H3K9me3 marks on the provirus, this effect was, however, clone-dependent as some clones did not reactivate after HUSH depletion (Tchasovnikarova et al., 2015). Indeed, Tchasovnikarova et al. (2015) found the HUSH complex to localize only on a specific group of endogenous genomic loci. Here again, the integration site appears critical as it determines the silencing ability of the HUSH complex.

Whether HIV latency is a byproduct of infection, a viral strategy to persist or on the contrary a cellular defense mechanism, remains to be determined. Overall, accumulating evidence pictures HIV latency as a multifactorial phenomenon that includes several control layers, that are probably overlapping and interacting but most importantly that are dynamic and responsive to environmental changes.

### Strategies to Address Latency: “Speak Now or Forever Hold Your Peace”

For more than 30 years, huge efforts have been mobilized in finding a cure for HIV, from vaccines to gene therapies, every available option has been investigated. To date, only one attempt at curing an HIV-1 positive patient revealed successful, namely the Berlin patient (Timothy Ray Brown) (Hütter et al., 2009). Diagnosed with an acute myeloid leukemia (AML), the Berlin patient received high doses of whole-body irradiation and two bone-marrow transplants from a homozygous CCR5Δ32 donor, providing resistance against HIV-1 infection. Since then, the Berlin patient showed no detectable signs of viral rebound for more than 5 years after cART interruption (Yukl et al., 2013). Unfortunately, attempts at reproducing this “sterilizing cure” on other seropositive patients failed, as viral rebound was observed as well as cases of drug- resistance and HIV tropism shift from CCR5 to CXCR4 co-receptor (Henrich et al., 2014; Kordelas et al., 2014). Latent reservoirs are thought to play a critical role preventing viral clearance by providing a constant pool of replicative competent viruses. Hence, two diametrically opposed strategies are envisioned to deal with what might be the last hurdle to HIV cure: purging the reservoirs, as proposed by the “shock and kill” strategy or on the contrary inducing a permanent viral control in the absence of therapy, in the case of a “functional cure” strategy.

### Shock and Kill

The “shock and kill” strategy was initially based on two observations: (i) the capacity of latent reservoirs to reactivate and shift from latent to productive infection, (ii) the cytopathic effect of viral replication and the killing of infected cells by the immune system (Archin et al., 2012a; Deeks, 2012). Activation of HIV expression in latent reservoirs is therefore expected to trigger elimination of HIV infected cells, while HIV dissemination would be prevented by cART. In this scope, several latency reversal agents (LRA) have been characterized including immune modulators [antibodies targeting immune checkpoints such as anti-PD-1 (Evans et al., 2018)], P-TEFb activators, protein kinase C (PKC) activators that induce NF-κB (bryostatin-1, prostatin and Ingenol) and inhibitors of chromatin-modifiers [such as HDAC inhibitors (HDACi) and histone methyltransferase inhibitors (HMTi)]. Although some of these LRAs show a slight increase of plasma RNA levels in clinical trials, none seems to effectively shrink the reservoir size (Elliott et al., 2014, 2015; Rasmussen et al., 2014; Søgaard et al., 2015; Spivak et al., 2015).

Besides the complexity of accurate quantification of the size of the reservoir, growing evidence indicate that given the heterogeneity of the latently infected cells, only a small proportion seems responsive to LRAs (Ho et al., 2013; Chen et al., 2017; Battivelli et al., 2018). Therefore, as with cART, combination of various LRAs would probably be required to achieve an effective “shock.” In addition, viral-induced cytopathic effect alone was found insufficient to induce the “kill” phase which implies that beforehand immune stimulation will be needed (Shan et al., 2012; Denton et al., 2014; Deng et al., 2015). Considered strategies to optimize reservoir elimination include broadly neutralizing antibodies, therapeutic vaccines and immune modulators, reviewed in Kim et al. (2018). However, multiplying drugs raises concern over toxicity and the impact of non-specific effects on gene expression (Walker-Sperling et al., 2016; Dental et al., 2017; White et al., 2018), thus requiring an equilibrium between efficacy and safety.

### Functional Cure

“HIV controllers” or “Elite controllers” are a rare population of HIV infected patients (<1%) who are able to spontaneously maintain their plasma viremia to almost undetectable levels. The precise mechanism responsible for this viral control is unknown but seems to result from several factors, including patient genetic backgrounds (Cockerham and Hatano, 2015). Based on the characteristics of these unique populations, the “functional cure” strategy aims at reaching durable remission in the absence of viral eradication. In fact, long-term remission was proved possible if therapy was initiated early during primary infection. Indeed, the “Mississippi child” born from a seropositive mother and who received cART 30 h after birth, remained with undetectable viral loads for 2 years after treatment interruption (Persaud et al., 2013). These results were confirmed with the VISCONTI cohort in which 14 patients reached long-term remission during a median period of 7 years after treatment interruption (Sáez-Cirión et al., 2013). Recently, a 12 years remission was also reported in a teen infected at birth and treated before 6 months of age (Frange et al., 2016).

Unfortunately, even under virological suppression, signs of ongoing viral replication were reported (Persaud et al., 2004; Fletcher et al., 2014; Lorenzo-Redondo et al., 2016). Remission can therefore last for long periods of time but eventually viral loads increase and therapeutic intervention is soon necessary. Consequently, recent strategies aim at providing more than viral control but rather a permanent inhibition of viral expression, thus preventing reservoirs’ reactivation through the establishment of a “deep latency” state.

Due to its crucial role in the efficient activation of HIV transcription, the viral protein Tat is often targeted by such strategies. For instance, an analog of cortistatin A, didehydro-cortistatin A (dCA), was shown to inhibit HIV Tat-dependent transcription by interacting with the TAR-binding domain of Tat, hence greatly reducing viral reactivation in response to stimulus (Mousseau et al., 2012, 2015). Another Tat inhibitor, Triptolide, is currently under clinal trial for its impact on reservoirs. Already used for rheumatoid arthritis, Triptolide was also reported to induce Tat degradation (Wan and Chen, 2014). Finally, this time through the inhibition of LEDGF/p75 interaction with the viral integrase, an interesting strategy proposes to avoid viral reactivation by directing HIV integration into transcriptionally inactive regions (Le Rouzic et al., 2013; Vranckx et al., 2016).

In any event, such therapies can only be delivered in combination with cART, at least during primary infection, and their potential in delaying or impeding viral rebound in patients remains to be demonstrated.

### What HUSH Antagonism by Vpx May Tell Us About HIV Cure Strategies?

In the context of “the shock and kill” strategy, the HUSH complex is a new factor to consider. It appears interesting to address the impact of HUSH complex inactivation on latently infected cells from patients, either through Vpx delivery or drug development. More importantly, HUSH antagonism by Vpx may have even deeper implications on our understanding of latency. Indeed, inactivation of an epigenetic regulator by a viral protein, further strengthens the idea of latency as a cellular defense mechanism, protecting genomic and proteomic integrity.

Hepatitis B virus x protein (HBx) was previously found to prevent H3K9me3 deposit by SETDB1 on the covalently closed circular HBV DNA (cccDNA) (Rivière et al., 2015), but the exact mechanism remains unknown. In our study, Vpx specifically binds and induces the degradation of an epigenetic regulator, Vpx thus identifies HUSH complex and the epigenetic machinery as part of the intrinsic immunity. The “shock and kill” strategy therefore may appear in opposition to the cellular defense mechanism with considerable risks due to the possible global epigenetic modifications. For instance, LINE-1 elements are also under the control of HUSH and transposition of LINE-1 can be responsible for disease as reviewed in Hancks and Kazazian (2016). On the contrary, “the block and lock” strategy, which aims at creating a state of “deep latency,” might be a better option to control viral expression, though with the drawback of a possible chronic immune activation. Finally, Axonal Charcot-Marie-Tooth (CMT) disease is a neurological disorder which was recently linked to the hyperactivation of the HUSH complex (Tchasovnikarova et al., 2017). This hyperactivation was shown to result from a mutation on the ATPase domain of MORC2 (R252W) (Tchasovnikarova et al., 2017). In case MORC2 is found to participate in the repression of HIV and that the sequence specificity of HUSH complex is proved, then it would be tempting to mimic such hyper activation through drug development, in order to force permanent silencing of exogenous elements including HIV.

## Author Contributions

GC and FM-G wrote this review, with, at the starting point, the thesis manuscript of GC. GC made the figures.

## Conflict of Interest Statement

The authors declare that the research was conducted in the absence of any commercial or financial relationships that could be construed as a potential conflict of interest.
